# Teaching STEAM in the shaolin staff program: ways to stimulate student engagement in learning

**DOI:** 10.3389/fpsyg.2023.1264985

**Published:** 2023-10-26

**Authors:** Haidong Chen, Feixue Rao, Ran Chen, Zhaowei Lin

**Affiliations:** ^1^School of Physical Education and Sports Science, South China Normal University, Guangzhou, China; ^2^School of Physics, South China Normal University, Guangzhou, China; ^3^Mechanical and Electrical Engineering Zhongkai University of Agriculture and Engineering, Guangzhou, China

**Keywords:** STEAM teaching concepts, shaolin staff, learning engagement, physics analysis, video analysis

## Abstract

With the advancement of technology and change in education concepts, applying STEAM pedagogy to physical education has become an innovative trend. This study investigates whether physics analysis based on STEAM concepts can increase junior high students’ participation in learning Shaolin Staff. Forty students were randomly assigned to experimental and control groups. The mean and standard deviation of age in the experimental group was 13.95 ± 0.83, and in the control group was 13.85 ± 0.81. The experimental group received STEAM teaching involving physics analysis, while the control group received conventional movement instruction. Student learning engagement was evaluated through questionnaires. Results are expected to show the experimental group being more engaged in learning. Introducing physics analysis may deepen understanding of related principles to movements. The study’s results found that the scores of motivation, attention, and intention to learn independently were significantly higher in the experimental group than in the control group (*p* < 0.01). The scores of interfering emotions were significantly lower in the experimental group than in the control group (*p* < 0.01), which verified that the STEAM teaching method could effectively improve learning engagement. This study promotes STEAM education in martial arts and provides insights on utilizing STEAM to boost student engagement.

## Introduction

STEAM education emphasizes the organic integration of science, technology, engineering, art, and other disciplines to provide a cross-disciplinary learning approach. This teaching method can stimulate students’ intrinsic motivation and enthusiasm for learning through a variety of ways, and STEAM education focuses on hands-on and inquiry-based project learning, allowing students to change from passive acceptance of knowledge to active participation in the process, giving full play to the subjectivity of learning (Wu, 2023). Research has shown that STEAM focuses on students’ creativity and imagination, encourages innovative practices, and allows trial and error. Project-making and art activities make learning fun. Furthermore, STEAM education provides a contextualized and immersive learning environment, applying knowledge to life situations and stimulating students’ emotional experience and engagement. In addition, the timely feedback mode of STEAM can bring a successful experience, satisfy students’ sense of achievement, and motivate them to continue learning (Belbase et al., 2022). It can be seen that the student-centered, participatory, and innovative learning elements of STEAM education can effectively stimulate motivation and enhance engagement in learning. This interdisciplinary approach teaches students about sustainable development while enhancing their understanding of related environmental issues and their ability to develop solutions. The interweaving of STEAM subjects with sustainability equips students with the knowledge and skills to comprehend and tackle the complex challenges of creating a just and ecologically sound future (Malecha, 2020).

STEAM education has gained traction recently, with increasing numbers of schools and teachers adopting this approach. As STEAM education proliferates, research into its implementation and impacts has grown. Song Naiqing et al. propose that assessment systems aligned with STEAM education principles could stimulate students’ intrinsic learning motivation. Integrating STEAM across curricula may cultivate key skills, including cognition, cooperation, innovation, and professional abilities. Well-designed assessments would allow students to identify their knowledge gaps, take initiative in their learning, and thus build endogenous motivation. This motivational force could potentiate the development of the diverse competencies needed to equip students for the future. Further research should investigate optimal assessment strategies to unlock the motivational and skills-development potential of STEAM education (Naiqing and Xin, 2021). Zhu Liming et al. propose that deep learning approaches inspired by the interdisciplinary framework of STEAM education could inform advances in core literacy, integrated curricula, and pedagogical innovations. Unlike technology-centric perspectives, STEAM-based deep learning promotes a humanistic style, prioritizing students’ emotional experiences and engagement during learning. Well-controlled studies are critical to elucidate the relative merits of humanistic, STEAM-based deep learning versus technology-focused methods. Such insights could refine emerging practices that effectively integrate STEAM and humanistic values, helping prepare students to navigate a complex world (Zhu Liming et al., 2023). Wenchao et al. propose integrating traditional cultural heritage within the interdisciplinary STEAM education framework. They argue that this synergy could enable the enduring transmission of cultural knowledge while advancing educational goals. The “interdisciplinary” ethos of STEAM could allow the holistic incorporation of traditional culture, infusing social responsibility, morality, and Chinese values. This may mitigate the utilitarian reputation of STEAM fields and foster deeper integration with the humanities. Further, student interest is critical for sustaining STEAM education (Wenchao et al., 2022). STEAM education for sustainable development takes this further by incorporating sustainability principles. This interdisciplinary approach teaches students about sustainable development while enhancing their understanding of related environmental issues and their ability to develop solutions (Hsiao and Su, 2021). For example, by integrating concepts of science, technology, and engineering with environmental ethics and nature conservation, students can gain holistic perspectives and skills to analyze complex sustainability challenges and develop innovative solutions (Yarime et al., 2012).

Yang et al. developed an elementary curriculum integrating STEAM and manufacturing concepts to foster interdisciplinary skills. This unified framework nurtures creativity, problem-solving, and student interest in STEAM fields. The authors highlight that stimulating self-efficacy through early manufacturing-STEM education may engage students and provide scaffolding to tackle complex real-world problems (Jia et al., 2021). Hsiao et al. explored the intersection of sustainability, STEAM education, and Virtual Reality (VR) technology in an experiential learning course. The study noted that combining STEAM with sustainability and VR technology to design an experiential learning course increased student motivation, satisfaction, and learning outcomes (Hsiao and Su, 2021).

Physical education is an important part of education. Integrating STEAM education concepts into physical education teaching can not only enable students to master sports skills and physical health knowledge but also cultivate comprehensive qualities such as scientific thinking, innovation, problem-solving ability, and teamwork so that they can have a more comprehensive development in physical education. While stimulating students’ interest in learning physical education courses, it opens students’ minds and further enhances teachers’ teaching effect. Integrating STEAM education concepts into physical education is an emerging teaching model that is gradually emerging at home and abroad, and there are more and more research results related to it. Lee examined the effects of STEAM-based physical education classes on students’ self-directed learning abilities and attitudes toward physical education classes related to alienation and avoidance of physical education through an experimental control method. The study showed that a physical education program incorporating STEAM education concepts positively impacted students’ attitudes and self-directed learning abilities in physical education classes, and he suggested integrating STEAM education with physical education (Lee, 2020). He also emphasized the need to apply dynamic pedagogical approaches and STEAM programs in physical education to address issues related to teacher instruction, student alienation, and avoidance of physical education (Lee, 2021). Yuan et al. explored the effects of continuous exercise on the positive psychological emotions of elementary school students by integrating STEAM education concepts under physical exercise. The current situation of physical exercise and the overall situation of positive psychological emotions of elementary school students were analyzed, and the role of physical exercise on positive psychological emotions of elementary school students was studied. Regular physical exercise can improve students’ physical and mental qualities and is essential for their comprehensive development (Yuan et al., 2022). The study of Iyakrus et al. concluded that the physical education teaching model established based on the STEAM education concept could effectively improve the physical fitness of elementary school students (Ramadhan, 2021).

It is clear from the above research that the STEAM education concept has emerged globally and is widely used in the current education sector. The concept combines science, technology, engineering, art, and math. It can cultivate students’ innovative and interdisciplinary thinking, stimulate their motivation and interest in learning, help them understand and apply knowledge more comprehensively, and cultivate comprehensive thinking and problem-solving skills. Incorporating STEAM education concepts into physical education teaching not only enables students to master motor skills and physical health knowledge but also improves their comprehensive qualities such as scientific thinking, innovation, problem-solving ability, and teamwork. So that they have a more comprehensive development in the field of sports. Domestic scholars have conducted fewer studies on integrating STEAM education concepts with physical education and even fewer studies on integrating traditional sports programs. Therefore, this study combines STEAM education concepts with the teaching curriculum of Shaolin Staff, a traditional Chinese sports program, to explore STEAM education concepts’ effects on students’ engagement in learning Shaolin Staff.

Many scholars in the above literature have explored the effects of integrating STEAM education concepts into teaching and learning on students’ independent learning ability, attitudes toward physical education programs, and mental–emotional and physical fitness through experimental design methods. Few studies combine STEAM education concepts with traditional Chinese sports programs, so the experimental designs and other research methods used in the above scholars’ studies can provide precedents and theoretical foundations for the design and conduct of the experiments in this paper.

Learning engagement refers to the degree to which students participate in the learning process’s physical, cognitive, and social dimensions. High levels of learning engagement increase student academic achievement, degree completion, and the creation of meaningful learning experiences. Learner engagement is an important instructional consideration in teacher education (Seo and Gibbons, 2019). This study focuses on five key dimensions of learning engagement: motivation, attention, intention to learn independently, satisfaction, and disturbing emotions. Motivation refers to students’ desire to participate in learning activities. Attention represents the focus and concentration on learning tasks. Intention to learn independently reflects students’ self-driven efforts to comprehend knowledge. Satisfaction indicates the fulfillment and pleasure gained through learning. Disturbing emotions refer to negative affective states like anxiety, boredom, and frustration during learning (Sun, 2022). Self-efficacy and self-directed learning abilities are closely related to learning engagement. Both self-efficacy and self-directed learning abilities foster a sense of ownership and empowerment in students’ learning experiences. When students believe in their capabilities and have the skills to guide their learning, they are more likely to become motivated, proactive learners who are fully engaged in their educational pursuits (Schunk and Zimmerman, 1994).

Personalized learning emphasizes that the learning process should begin with the learner and that participation should drive decision-making. Implementing and assessing personalized learning environments give learners more voice, promoting engagement and better learning outcomes. Thus, learning engagement can be used to view the learner experience, which enriches the understanding of the language of learner performance, skills, and competencies. Understanding the complexity and multidisciplinarity of learner engagement can be achieved by exploring the development of personalized learning environments and applying ideas from social media (Ballard, 2013). Learner engagement is strongly associated with important educational outcomes such as academic achievement and satisfaction. While some research has been conducted on learner engagement in blended learning environments, there is a lack of consistency and specificity in the definition, operationalization, and measurement of engagement. Therefore, developing a framework for defining, modeling, and measuring factors of learner engagement is critical to determining whether changes in instructional methods increase engagement. The study reviews existing literature on learner engagement and identifies constructs relevant to blended learning. The study proposes a possible conceptual framework for engagement, including cognitive and affective metrics, and provides methods for measuring these metrics in a technology-mediated learning environment (Halverson and Graham, 2019). The importance of engagement in learning has received much attention in education. Student engagement is closely related to educational outcomes, such as academic achievement, learning satisfaction, and school retention. Research has shown that creating interactive, personalized learning environments promotes student engagement. Teachers play a key role in the teaching and learning process, and they should use various teaching strategies to encourage active student participation and provide support and feedback. In addition, schools and education policymakers should also pay attention to learning engagement and provide resources and support to improve the learning environment to stimulate students’ motivation and interest in learning (Carini et al., 2006). Overall, learning engagement significantly impacts student achievement and learning experiences.

The staff, the most representative long weapon of Chinese martial arts, has much room for development. For thousands of years, it has been the most adaptable of Chinese martial arts instruments (Liu, 2014). An ancient martial arts proverb says, “The staff is the leader of all martial arts (Tang, 2008).” It is in this sense that it is said. On the other hand, the staff is also the most representative and central instrument of Shaolin Kungfu. The staff has played an important historical and cultural role in different Shaolin Kung Fu development periods. The unique guardian of the Shaolin Temple, King Jinnaluo, was famous for using the staff, and the historical legend of “Thirteen Monks with Staffs Saving Tang “also centered on using the staff by monks and soldiers. In the Ming Dynasty (1,386–1,644), when the development of Shaolin martial arts was in a subversive stage, the Shaolin staff became the most representative element of Shaolin Kung Fu in the world of martial arts (Shahar, 2001). At that time, Shaolin monks and soldiers were ordered to be recruited to the southeast coast to fight against the Japanese and protect the country. One of the basic techniques used was the Shaolin Staff Technique. Yuanyi, in his great book on military science, Wu Bei Zhi, has a paragraph stating that “all martial arts are based on the staff, and the staff is based on Shaolin” (Mao, 1984).

The inheritance and innovation of Shaolin Staff teaching methods are crucial to promoting the modernization and social value of this traditional martial art. Integrating STEAM concepts can achieve this goal by making learning more engaging and interdisciplinary. Student participation is vital for engagement in the STEAM teaching process. Therefore, this study assesses students’ interdisciplinary knowledge and learning engagement to reflect the quality of STEAM-integrated Shaolin Staff instruction. Analyzing physics principles cultivates systemic thinking to comprehend complex issues like sustainability. Assessing learning engagement provides key insights into the effectiveness of applying STEAM to modernize Shaolin Staff teaching while making it relatable to students’ development needs. This underscores the importance of participatory STEAM instruction for inheriting and innovating traditional martial arts education.

This study is framed by the theory of learning engagement (Halverson and Graham, 2019), which emphasizes active participation and student-centered learning. The degree of engagement is associated with academic outcomes. The implementation of STEAM education is grounded in constructionist learning theories (Morado et al., 2021), which posit that learning occurs most effectively when students construct their understanding through designing and creating things. Video analysis technology draws on personalized learning theories (Peng et al., 2019), highlighting customized feedback to meet individual learner needs. These theories jointly informed the instructional design and analysis of results.

We are developing a series of traditional martial arts programs integrating STEAM to achieve this goal. The aim is to fully develop students’ knowledge, abilities, and literacy at the K-12 level. This paper reports the results of our first round of development and answers the following questions: (a) How to integrate STEAM concepts into the curriculum framework based on STEAM teaching and learning? (b) How do we develop a curriculum based on the framework? (c) How to assess the effectiveness of the developed curriculum? We expect to improve the Shaolin Staff curriculum’s learning experience and outcomes by focusing on student engagement in learning.

## Research objects and methods

### Objects of study

A non-random sampling method was used in this study. The following requirements were met: the study subjects had to be in the second year of junior secondary school. This means they should be in the second academic year of the secondary education system, usually between the ages of 13 and 14. The study population consisted of both boys and girls. It has been shown that a small class size is more suitable for blended learning programs like STEAM because it is more conducive to teacher-student interaction. In small class teaching in China, a class usually has no more than 25 students (Jyun, 2021).

## Research methods

### Experimental method

**Figure 1:**
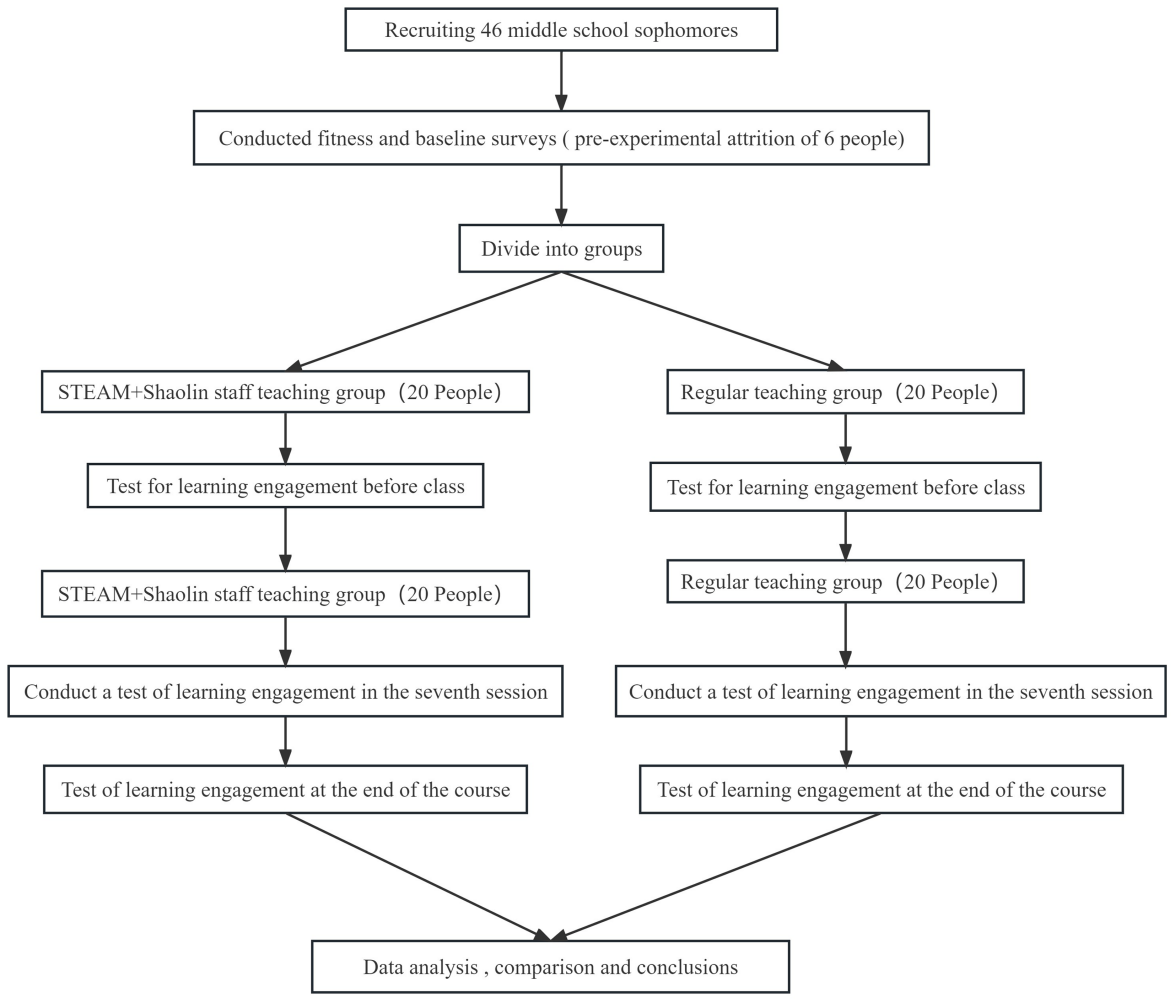
Flow chart of instructional design.

The experimental group was taught martial arts by introducing the STEAM teaching concept, and the control group was taught traditional martial arts. The teaching process was divided into three main parts, i.e., the preparation part (5 min), the basic part (35 min), and the ending part (5 min), and the same teacher led the practice for a total of 14 lessons. In the pre-experimental period, the POLAR TEAM2 table and the K4B2 cardiopulmonary function tester were applied to test the exercise intensity of the Shaolin Staff Technique. It was made to reach the medium intensity level and medium intensity aerobic exercise. The athlete’s respiratory heart rate increased significantly, and the heart rate reached (170-age) ± 10–20 beats. At this time, the subjective feeling is that the respiration and heartbeat are accelerated, slightly sweating, and can speak but not sing (Shanxi Sports Science Institute, 2021). The moderate intensity in this experiment was controlled at (Max HR) 60–70%. The heart rate was monitored in real time for experimental and control groups. Wearing a heart rate monitor controlled their heart rate at a moderate intensity level. The exercise intensity can be controlled in the experiment by adjusting the time between exercises and intervals, the center of gravity, and the height of movements during exercises so that the subjects sweat slightly without feeling fatigued. Parents or guardians of all the participants in this study signed an informed consent form to ensure that the study complied with ethical norms. There were ten boys and ten girls in each group of this experiment. There were 20 cases in the experimental group and 20 in the control group. Consent to participate in this study should be obtained from the study subjects. Patients with contraindications to exercise were excluded. Subjects who were unwell on their way to the experiment and those who needed better compliance and did not comply with the experiment were excluded. Figure 1 shows the design flow of this experiment.

### Framework for developing an integrated STEAM program

#### Course overview and goals

Introduce the background and goals of the course, emphasizing the importance of STEAM teaching concepts in increasing student engagement in learning. Articulate the goals of the course, including increasing student interest, motivation, and engagement in the Shaolin Staff program, as well as fostering their scientific literacy and skill development.

#### Science module

Introduces the principles of human kinematics and mechanics and explores their relevance to staff movements. Analyze the effects of staff fighting on the body and study energy transformation and force. Explore the relationship between staff skills, anatomy, physiology, and motor control. Conduct hands-on activities that allow students to apply scientific principles to optimize staff handling movements.

#### Technology module

Quantitatively analyzing and improving staff movements using video analysis software. Record and analyze individual staff-fighting demonstrations using video capture and editing techniques. Use mobile apps and online resources to access information about martial arts history and techniques.

#### Art module

Appreciate ancient Shaolin staff fighting songs and drawings and create routines that express Shaolin culture and philosophy to demonstrate the beauty of staff fighting. Students are encouraged to learn traditional Shaolin staff fighting performances and movement choreography to demonstrate grace and fluidity of movement.

#### Mathematics module

Analyze the angles and strength of staff movements involving concepts of geometry and trigonometry. Calculate the speed and acceleration of staff movements using kinematics and dynamics formulas.

#### Assessment and feedback approach

Utilize an array of effective assessment methods, encompassing practical performance evaluations, interviews, and questionnaires. The Learning Engagement Scale was employed to comprehensively evaluate students’ engagement, including motivation, attention, intention to learn independently, satisfaction, and disturbing emotions (Sun, 2022). The scale’s internal consistency reliability (Cronbach’s alpha) was established at 0.81, ensuring measurement accuracy. Employ questionnaires to gather data on students’ learning engagement and gage their level of involvement and interest in the course. Implement a consistent cycle of instructional feedback from educators and students to continually enhance and refine course content and instructional techniques.

#### Integration of STEAM framework and interview insights

Our developmental framework integrates STEAM teaching principles by fusing science, technology, art, and math into the Shaolin Staff curriculum. This strategic integration elevates student learning engagement by applying physics analysis and video analytics. After the experiment, we conducted semi-structured interviews with 20 participants randomly selected from the STEAM teaching group. The primary aim was to gather qualitative insights into their learning journey and perceptions of the STEAM-incorporated Shaolin Staff course. The interview format encompassed open-ended questions strategically designed to uncover students’ perspectives regarding the amalgamation of science, technology, art, and math within the curriculum and the potential impact on their learning engagement.

### Course design of Shaolin staff technique

The course covers several principles of physics:

1. Rotational Moment: The rotational moment is the force acting on an object that causes the object to rotate around a fixed axis. Its magnitude is equal to the product of the force and the force arm, which is the perpendicular distance between the force and the axis of rotation. The rotational moment determines the speed and direction of rotation of the object.
2. Moment of Inertia: The moment of inertia is the property of an object that resists changing its rotation state. It is related to the object’s mass distribution and its axis’s position. The larger the moment of inertia, the more difficult it is for the object to change its rotation state. The moment of inertia can be used to describe the response of an object to a rotational moment.
3. Angular Velocity: Angular velocity is the rate of change of angle per unit of time when an object is rotating about an axis. It indicates the speed of rotation and is expressed in radians per second. The greater the angular velocity, the faster the object rotates.
4. Linear Velocity: Linear velocity is the speed of a point on an object along a trajectory as it rotates. It is related to the angular velocity and the distance from the axis of rotation to that point. Linear velocity is a physical quantity that describes the difference in velocity at different points on a rotating object.
5. Balance Lever: A balance lever is a rigid rod supported by a fulcrum with forces of different magnitudes or directions acting on either side. The principle of a balanced lever is based on the law of levers, which states that the left side moment equals the right side moment, making the lever balanced. This means that a balanced lever will remain at rest when the force on the left multiplied by its distance to the fulcrum is equal to the force on the right multiplied by its distance to the fulcrum.
6. Interaction Force: Interaction force is the force exerted by two objects on each other. According to Newton’s third law, interaction forces always occur in pairs and are equal in magnitude and opposite direction. When one object exerts a force on another object, the other object also exerts a force of equal magnitude and opposite direction on the first object. These forces are the result of the interaction between two objects (see Table 1).

**Table 1:** Course design of Shaolin Staff technique.

Subject	Action	Content	Lesson	Physical principles
Fighting position	Zhongsiping position (中四平势)	Stand upright, holding the staff in the right hand to keep it straight. Turn to the left side with the right foot as the axis, and at the same time, step back with the left foot, forming a Gongma stance, with the distance between the feet slightly greater than the shoulder and the center of gravity of the body falling between the two feet. The toes of the right foot are slightly inwardly buckled toward the front, the toes of the left foot are toward the right front, and the heel is lifted; the horizontal distance between the front and back feet is about half a foot; at the same time, the left-hand slides downwards along the staff, and grips it to the end of the staff, the right-hand grips the staff and presses it forward and downwards, with the two arms slightly curved, and the distance between the hands is somewhat wider than that of the two feet; the upper body stays straight, the chest and the stomach are contained, the two shoulders are sunk to the lower part of the body, and the front end of the staff is slightly lifted, with the eyes looking at the front.	1	Balance stability, the center of gravity adjustment
footwork	Xiaogongbu (小弓步)	Turn hips with the left foot in the stirrups so that the front of the knee is perpendicular to the ground and the front of the body is facing forward.	1	Interaction forces, the center of gravity adjustment
	Gongbu (弓步)	Stomp the ground with the back foot and take a big step forward with the front foot while bending at the knee, with the thigh nearly horizontal and the knee perpendicular to the toes.	1	
	Tibu (提步)	The right foot stirs the ground, the center of gravity shifts back to the left leg, the right foot is quickly lifted to the rear, and the left leg is assisted to stir straight. Currently, the left leg independently supports the body, the right leg is lifted backward in the air, the center of gravity is transferred from the left leg to the waist and abdomen, and the body is tilted forward.	1	
Offensive technique: Zha	Pinzha (平扎)	The left foot stirrups and turns to the right, quickly driving the left side of the waist and hips to the right so that the body is facing forward; the left shoulder is sent on at the same time as the left arm holding the soldier ahead stabbing, the right arm is quickly raised to assist in the forward delivery of the right-hand does not move to ensure that the staff from the right-hand slides through smoothly.	1	Rotational torque, moment of inertia, linear velocity
	Shangzha (上扎)	Twist the left foot to the right, quickly drive the left side of the waist and hips to the right so that the body is facing forward, the left shoulder to the front at the same time as the left arm forward and upward stabbing, the right arm quickly raised to assist the forward and upward, the right-hand does not move, to ensure that staff from the right-hand slides through smoothly.	1	
	Xiazha (下扎)	The left foot stirrup is twisted to the right, quickly driving the left side of the waist and hips twisted to the right so that the body is facing forward; the left shoulder is sent forward at the same time that the left arm is stabbed forward and downward, the right arm is quickly raised to assist the forward and downward, the right-hand does not move to ensure that the staff from the right-hand slides through smoothly.	1	
	single-handed zha (单手扎)	The left foot stomps the ground to twist to the right, quickly driving the left side waist and hips to twist to the right; the left shoulder is sent out to the front to the maximum extent at the same time the left arm jabs out to the front, at the same time the right hand detaches from the staff and quickly extends out to the back to assist the body to twist to the right.	1	
Offensive Technique: Li	Xia Pi (下劈)	The left foot slightly stirrups the ground, the left side of the waist and hips and shoulders quickly twisted to the right so that the front of the body facing the front, the left hand forward and upward lifting equipment, and the forehead at the same height position, at the same time, the right hand quickly along the equipment downward slide, so that the right hand is located in the head is directly above. Then the waist and hips quickly to the left twisted, the right hand sliding the handle forward to the downward pressure, the left hand is also downward pulling and pressure, so that the equipment downward cleaves to the horizontal position, arms upward force to inhibit the continuation of downward movement of the equipment.	1	Rotational torque, moment of inertia, angular velocity, linear velocity
Advanced Offensive Techniques	Immortal sits in a cave posture (仙人坐洞势)	The center of gravity is shifted back to the left leg, the body sits back, the end of the staff is pressed against the left side of the waist and abdomen, the staff is pointed toward the opponent, and then one hand is zipped out violently.	1	Balance stability, the center of gravity adjustment
	Jiashan posture (夹衫势)	The right hand violently sends the staff backward. Both hands simultaneously slide forward to grasp at the front half of the staff; the left arm holds the staff under the armpit, the right hand will nearly grasp at the front end of the staff, and the left foot steps forward upward and quickly approaches.	1	
Defensive techniques	Upper right block (右上路格挡)	The body is turned slightly to the right; the right arm is bent at the elbow, the right hand slides slightly under the handle and recovers toward the right front of the chest, and the left hand is then pushed slightly forward and downwards so that the upper part of the staff protects the head.	1	leveraging
	Left center block (左中路格挡)	The body is turned to the left; the right arm is bent at the elbow; the right hand slides the handle slightly upwards and recovers toward the left front of the head, and the left-hand moves forward to be in a perpendicular position to the right hand so that the staff is guarded on the left side of the torso.	1	
	top-shelf block (上架)	Turn right with left foot on the ground, turn body naturally to the front, and raise both arms forward and upward simultaneously so the staff is laterally parallel to the ground and high above the head.	1	

**Figure 2:**
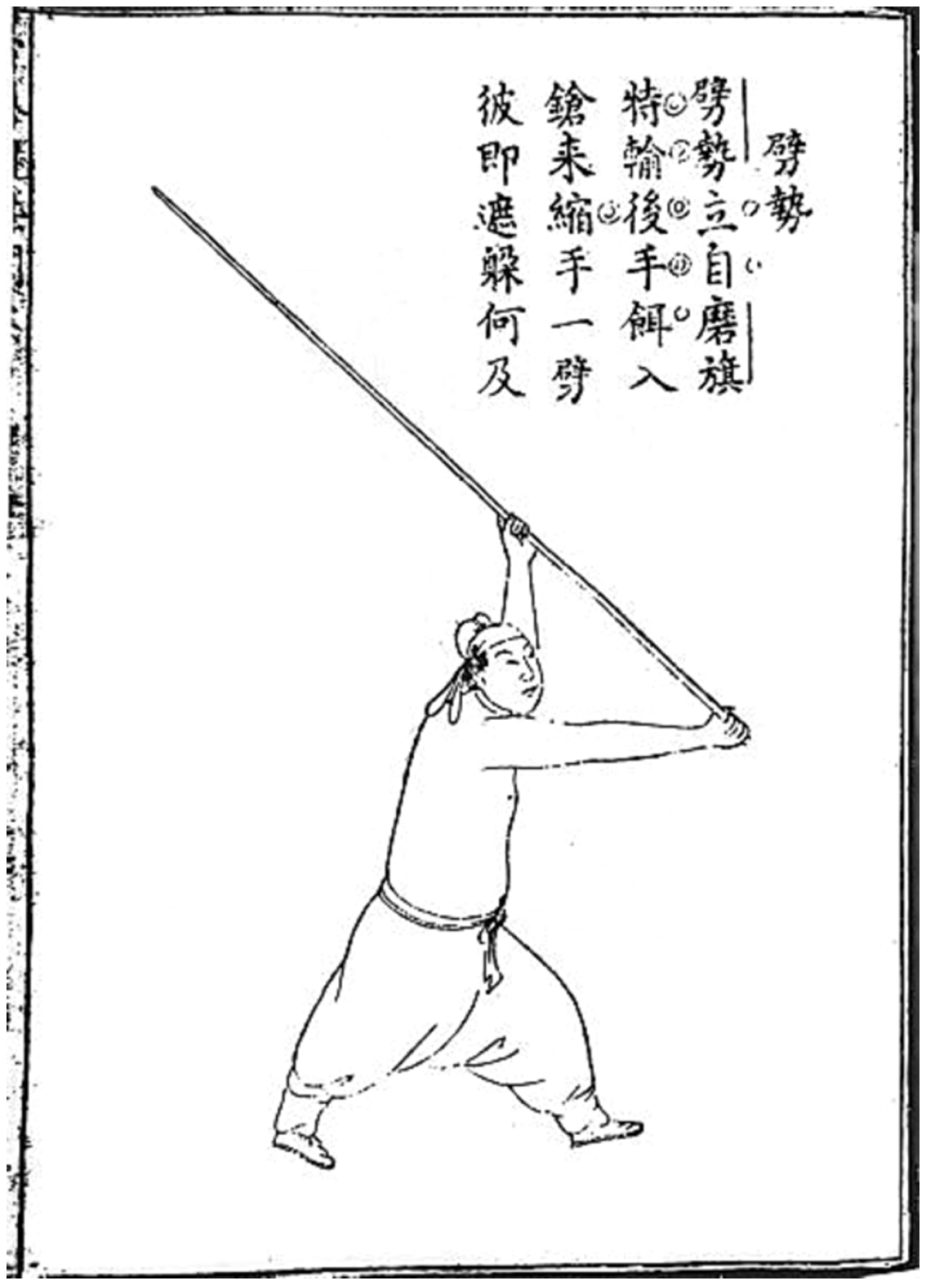
Xiapi posture of the Ming Dynasty “Shaolin Staff Method Elaboration” (Ma and Ma, 2015).

**Figure 3:**
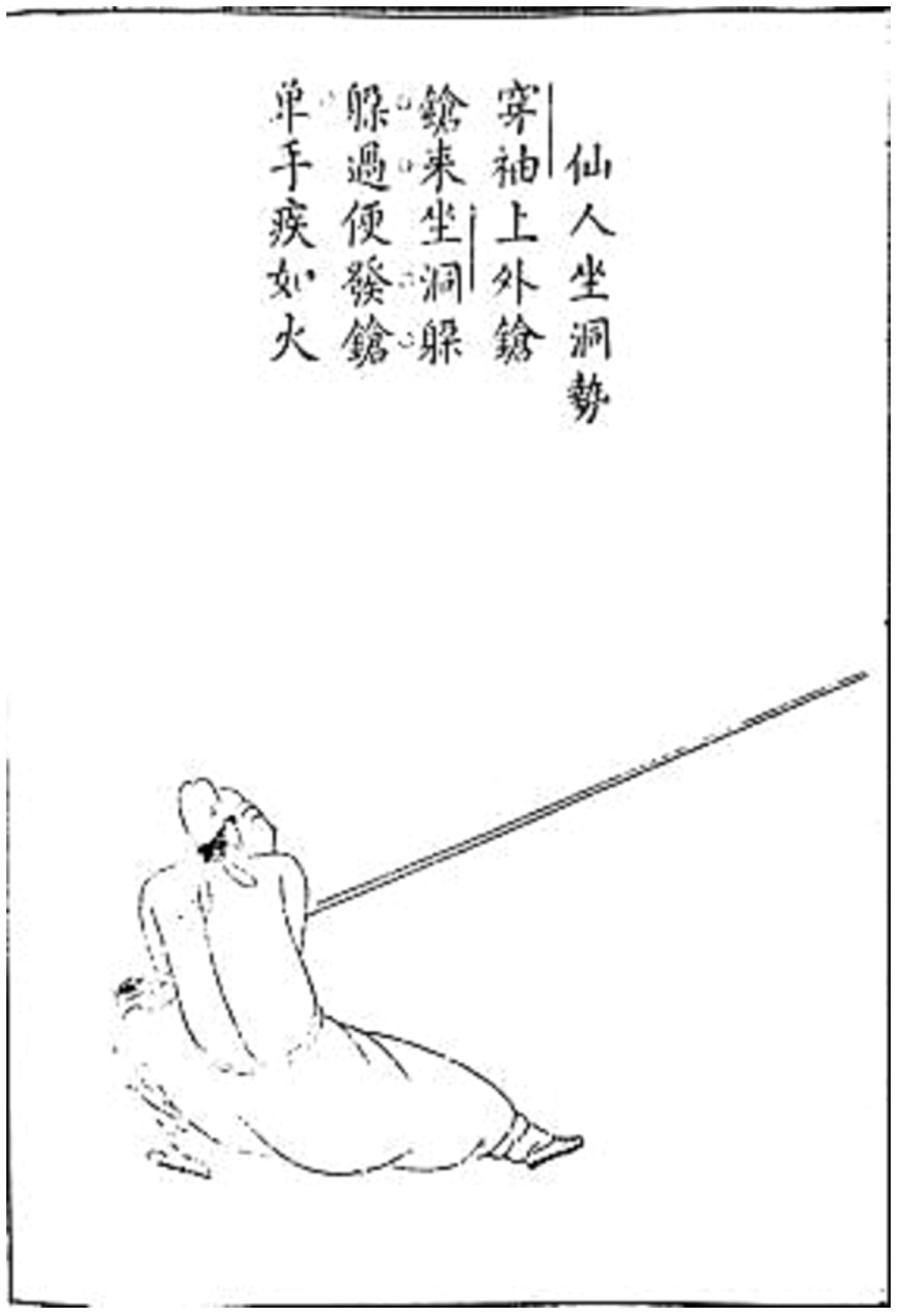
Immortal sits in a cave posture of the Ming Dynasty Shaolin Staff Method Elaboration (Ma and Ma, 2015).

In addition, the content we selected for teaching Shaolin Staff originated from the book “Explanation of Shaolin Staff Technique” by Cheng Zongyou, a famous martial arts master in the Ming Dynasty. In order to realize the characteristics of Arts in the STEAM teaching concept, we also set the contents of the drawings and staff tips in the staff score in the teaching of the corresponding technical movements, respectively. This enables students to understand the historical origins of the Shaolin Staff Technique and improves their ability to recognize and discover beauty (as shown in Figures 2, 3).

### Teaching cases

#### Description of the Xiapi (下劈)

The left foot slightly stirs the ground, and the left waist, hips, and shoulders are quickly twisted to the right so that the front of the body faces the front. The left-hand raises the staff forward and upward to a position at the same height as the forehead. At the same time, the right hand quickly slides down the staff so that the right hand is directly above the head. The waist and hips are then quickly twisted to the left, and the right-hand slide handle is pressed downward. The left hand pulls down and presses down so the staff splits horizontally. The arms are then pushed upward to inhibit the downward movement of the staff.

This movement can be analyzed in three stages:

1. Initial state: the right foot is in front, and the left is behind in a slight stirrup. The front of the body is slightly to the left, arms down, holding the staff.
2. Stage 1: the left side of the waist, hips, and shoulders are twisted to the right, utilizing the legs and lower back muscles to exert force and give themselves a rotational torque. This torque can be described by Newton’s law, acting on the center of mass of the body with the magnitude:

(1) M=J∗a

M is torque, J is the moment of inertia, and a is the angular acceleration. The moment of inertia is a physical quantity that describes the ease with which an object can rotate, and it can be calculated using the following equation:

(2) $$J=m*r^{2}$$

Where m is the object’s mass and r is the distance from the axis of rotation to the center of mass.

At the same time, the left-hand raises the staff forward and upward to a position at the same height as the forehead. The right hand quickly slides down the staff to position the right hand directly above the head. During this process, the left arm will be lifted faster than the right arm slides down, and the force arm becomes longer; the body’s rotational inertia is also changing.

During this process, the lower limb muscles must be controlled to support the body weight and maintain balance. At the same time, the involvement of muscles such as the low back musculature and the rectus abdomens plays a key role in the steering of the body. The lifting motion of the left arm is achieved through the strength of the scapular girdle and upper arm muscles. The sliding of the right hand, on the other hand, requires the corresponding joints of the shoulder, elbow, and wrist to work in tandem to control the accuracy and speed of the movement.

1. Stage 2: the waist and hips are quickly twisted to the left, the right-hand slide handle is pressed forward and downward, and the left hand is pulled downward and pressed so the staff splits downward when it reaches a horizontal position. The velocity of the endpoint of the staff changes from $\vec{v_{1}}$ to $\vec{v_{2}}$ with the velocity formula:

(3) $$\vec{v_{1}}=\vec{\omega_{1}}\times \vec{R_{1}}$$

(4) $$\vec{v_{2}}=\vec{\omega_{2}}\times \vec{R_{2}}$$

Where $\vec{\omega_{1}}$ and $\vec{\omega_{2}}$ are the angular velocity before and after action, $\vec{R_{1}}$ and $\vec{\;R_{2}}$ are the radius before and after action, respectively.

This process involves muscle contraction and relaxation to maintain balance in the body’s tilting and the staff’s movement. At the same time, the sliding speed of the staff must be allowed to accelerate at an even rate to increase the force on the target.

1. Stage 3: the arms are powered upward to inhibit the continued downward movement of the staff. The main function of this phase is to counteract the effect of gravity on the staff and ensure that the staff does not continue to fall downward after the downward chop has reached the designated position. In this process, a reverse force equal to the gravity of the staff needs to be applied, which can be calculated using the following formula:

(5) $$\left|\vec{F_{1}}\right.+\left.\vec{F_{2}}\right|=m_{器械}*g$$

This process requires a synergy of muscles throughout the body to stabilize the balance of the body and control the staff through the resistance of the arms and force generation of the legs.

**Figure 4:**
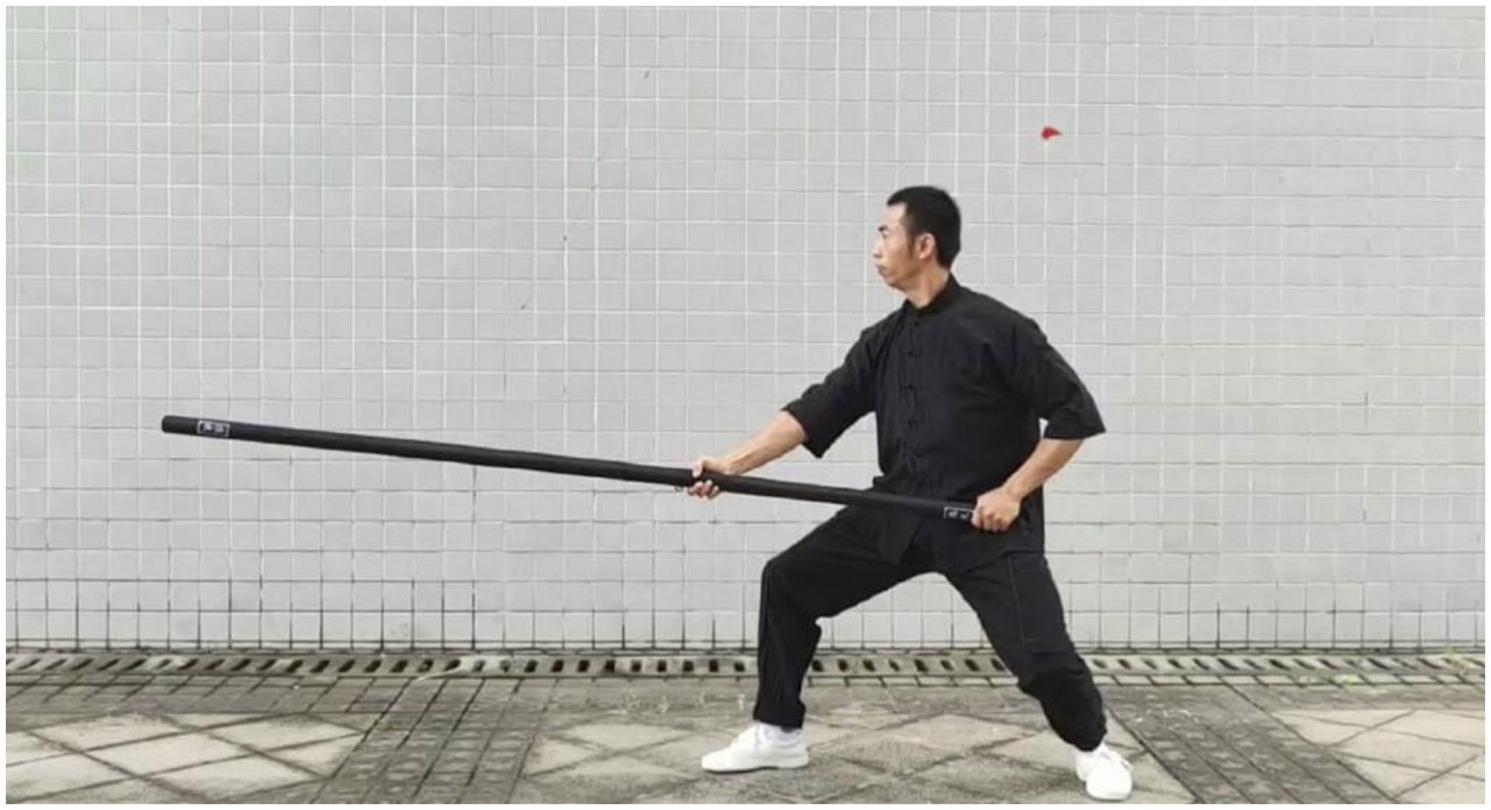
Initial state of Xiapi.

**Figure 5:**
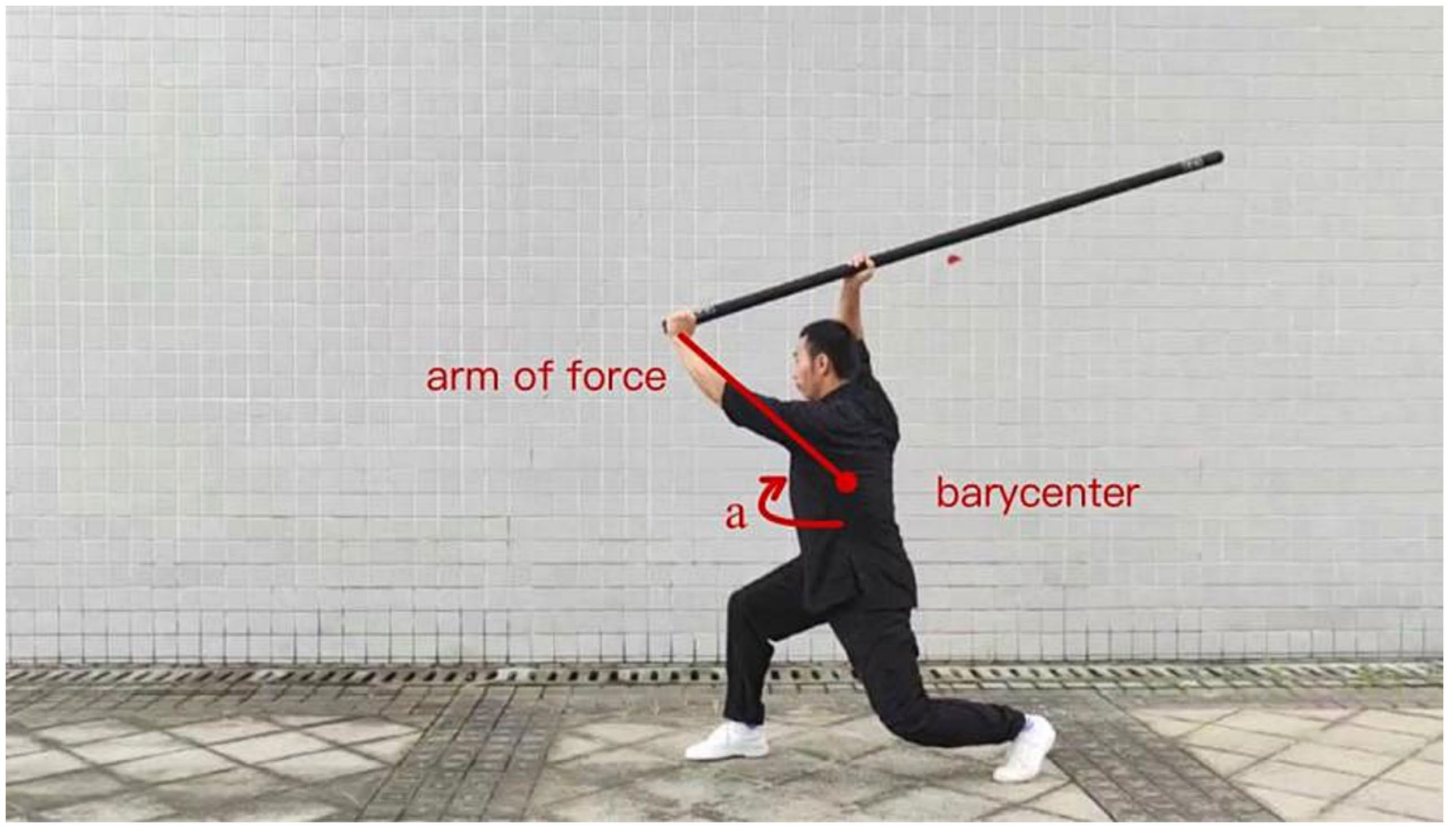
Stage 1 of Xiapi.

**Figure 6:**
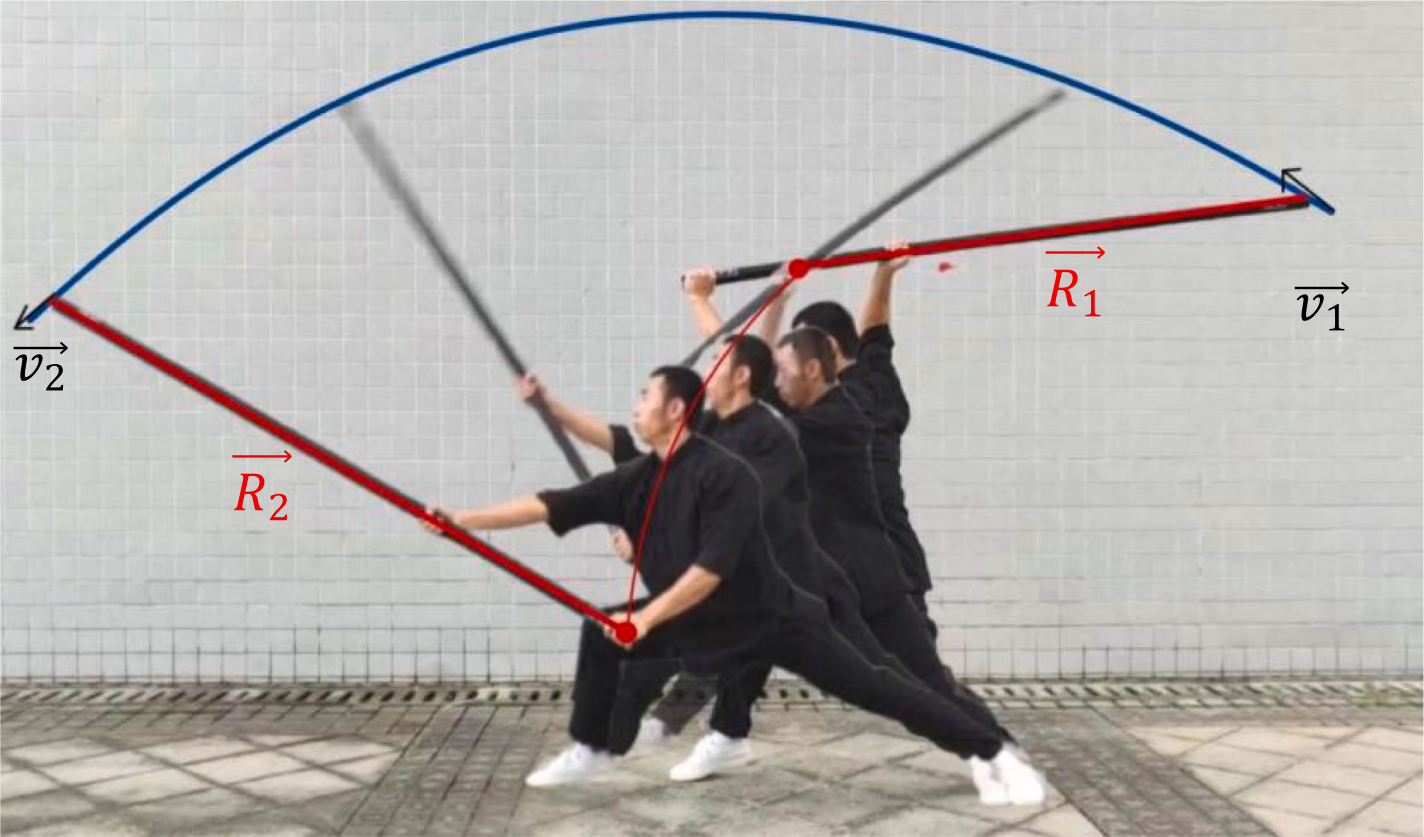
Stage 2 of Xiapi.

**Figure 7:**
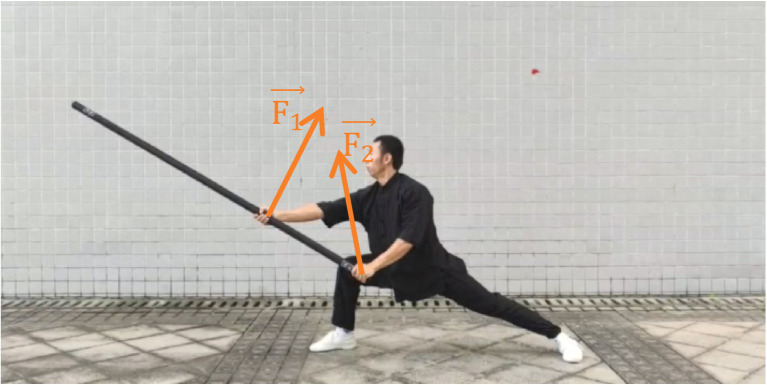
Stage 3 of Xiapi.

The main mechanical concepts involved in the downward chop are rotational moment, balanced leverage principle, interaction force, muscle control, etc. Strong muscle strength and body control are required to maintain good balance and arm accuracy. At the same time, one needs to master the coordinated movement of the scapular girdle and upper arm muscles to achieve smooth and powerful movements (Figures 4–7).

## Findings of the study

### Students’ engagement in learning

The five dimensions of learning engagement examined were motivation, attention, intention to learn independently, satisfaction, and disturbing emotions. The learning engagement scores collected from the questionnaire survey were compared and analyzed between the two groups before and after the experiment. The chi-square test was used for count data. The count data were calculated to be consistent with normal distribution, which repeated measures and group simple effects tests can test. As shown in Table 2, there is an interaction effect between different interventions and intervention time in the dimensions of motivation, attention, intention to learn independently, satisfaction, and disturbing emotions.

**Table 2:** Comparison of experimental and control groups’ learning engagement scores before and after the experiment (X ± SD).

Factor	Group	(M±SD)			Group main effect			The number of measurements main effect			Interaction effect of the number of measurements and group		
		Baseline	Three weeks	Six weeks	F0	bias η^2^0	P0	F1	bias η^2^1	P1	F2	bias η^2^2	P2
Learning Motivation Dimension	T	2.37±0.40	3.19±0.46	4.06±0.31	11.39	0.23	0.02^*^	99.95	0.73	<0.01^**^	16.84	0.31	<0.01^**^
	C	2.55±0.60	2.82±0.44	3.26±0.33									
Attention Dimension	T	2.41±0.40	3.01±0.41	3.99±0.25	16.91	0.31	<0.01^**^	94.76	0.84	<0.01^**^	24.82	0.40	<0.01^**^
	C	2.48±0.47	2.72±0.38	3.07±0.38									
Self-Directed Learning Intention Dimension	T	2.46±0.51	3.15±0.21	3.92±0.22	27.55	0.42	<0.01^**^	78.40	0.67	<0.01^**^	11.47	0.23	<0.01^**^
	C	2.48±0.52	2.67±0.45	3.14±0.36									
Satisfaction Dimension	T	2.31±0.44	3.14±0.39	3.95±0.36	9.89	0.21	0.03	89.46	0.83	<0.01^**^	29.72	0.44	<0.01^**^
	C	2.58±0.55	2.81±0.34	3.09±0.33									
Disturbing emotional dimensions	T	3.47±0.30	2.97±0.34	2.21±0.30	7.54	0.17	<0.01^**^	145.26	0.89	<0.01^**^	14.60	0.28	<0.01^**^
	C	3.45±0.29	3.18±0.31	2.72±0.36									

“*” indicates *p* < 0.05; “**” indicates *p* < 0.01.

**Figure 8:**
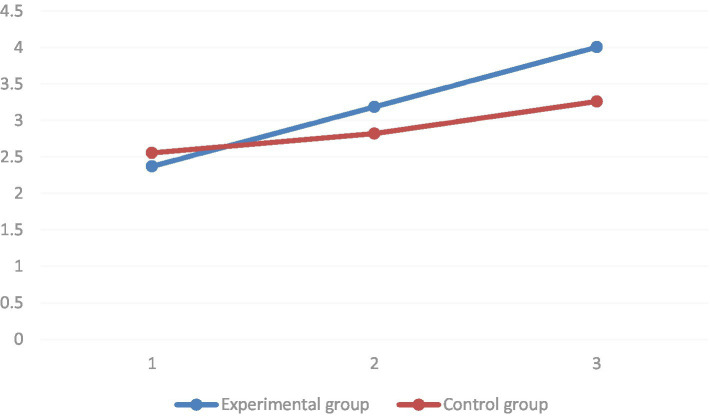
Comparison between experimental and control groups before and after the experiment of estimated marginal means of learning motivation dimensions.

Regarding the learning motivation dimension, it was found by multiple comparisons that the mean values of the learning motivation dimension of the pre-test, the mid-test, and the post-test of the learning motivation dimension increased in the order of significance (*p* < 0.01) in the group where the STEAM teaching concept was introduced. In the group using conventional teaching, the mean value of the learning motivation dimension of the pre-test was significantly higher than that of the post-test (*p* < 0.01). However, it was not significantly different from the mid-test (*p* = 0.093). The group simple effects analysis results showed that in the baseline, there was no significant difference in the mean values of the learning motivation dimension between the two groups (*p* = 0.26). In both the mid-test (*p* = 0.01) and post-test (*p* < 0.01), the mean values of the motivation to learn dimension were significantly higher in the STEAM teaching group than in the regular teaching group (as shown in Figure 8).

**Figure 9:**
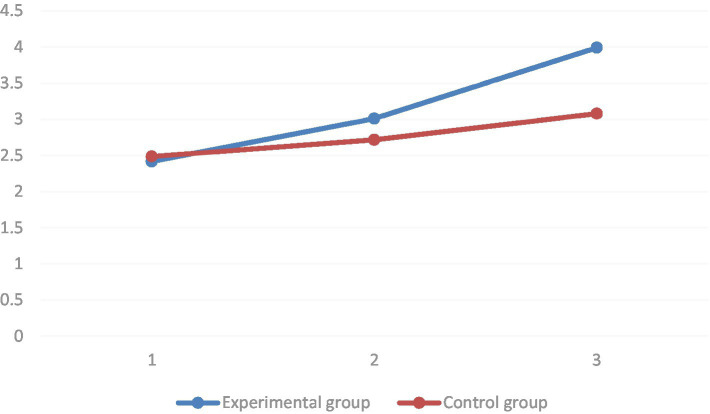
Comparison of the experimental and control groups before and after the experiment of estimating the marginal mean of the attention dimension.

In terms of attention dimension, it was found by multiple comparisons that in the group where the STEAM teaching concept was introduced, the mean values of attention dimension of pre-test, mid-test, and post-test of attention dimension increased sequentially, and all of them reached the level of significance (*p* < 0.01). In the group with conventional teaching, the mean value of the attention dimension of the pre-test was significantly lower than that of the post-test (*p* < 0.01). However, it was not significantly different from the mid-test (*p* = 0.062). The group simple effects analysis results showed that in the baseline, there was no significant difference in the mean values of the attention dimensions between the two groups (*p* = 0.63). In both the mid-test (*p* = 0.02) and post-test (*p* < 0.01), the STEAM instruction group had significantly higher mean values of attention dimensions than the regular instruction group (as shown in Figure 9).

**Figure 10:**
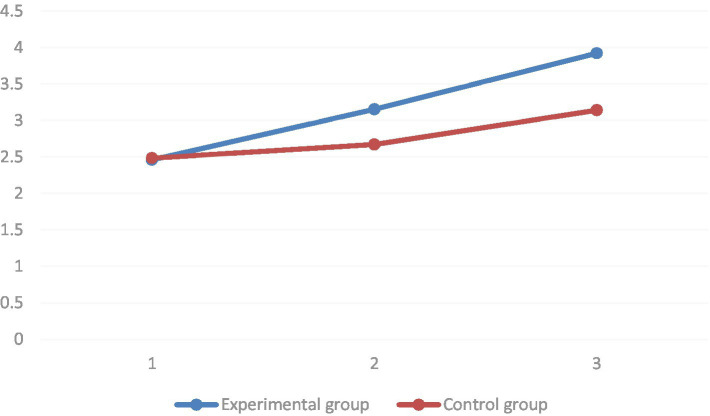
Comparison between the experimental and control groups before and after the experiment of estimating the marginal mean of the dimension of the autonomous learning intention.

Multiple comparisons found the independent learning intention dimension. In the group where the STEAM teaching concept was introduced, the mean values of the independent learning intention dimension of the pre-test, the mid-test, and the post-test of the independent learning intention dimension increased sequentially. All reached the level of significance (*p* < 0.01). In the group with conventional instruction, the mean value of the attention dimension of the pretest was significantly lower than that of the posttest (*p* < 0.01). However, it was not significantly different from the middle test (*p* = 0.23). The group simple effects analysis results showed no significant difference in the mean values of the independent learning intention dimension in the baseline between the two groups (*p* = 0.88). In both the mid-test (*p* < 0.01) and post-test (p < 0.01), the mean values of the attention dimension were significantly higher in the STEAM teaching group than in the conventional teaching group (as shown in Figure 10).

**Figure 11:**
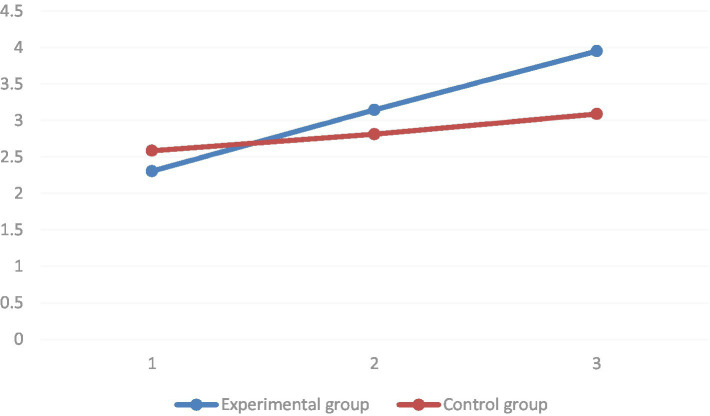
Comparison between experimental and control groups before and after the experiment of estimating the marginal mean of satisfaction dimension.

In terms of satisfaction dimension, it was found by multiple comparisons that the mean values of satisfaction dimensions of pre-test, mid-test, and post-test satisfaction dimensions of satisfaction dimensions increased sequentially in the group where the STEAM teaching concept was introduced, and all of them reached the level of significance (*p* < 0.01). In the group with conventional teaching, the mean value of the satisfaction dimension of the pre-test was significantly lower than the post-test (*p* < 0.01) but not significantly different from the mid-test (*p* = 0.14). The group simple effects analysis results showed that in the baseline, there was no significant difference in the satisfaction dimension means between the two groups (*p* = 0.86). In both the mid-test (*p* = 0.07) and post-test (p < 0.01), the STEAM teaching group had significantly higher satisfaction dimension means than the regular teaching group (as shown in Figure 11).

**Figure 12:**
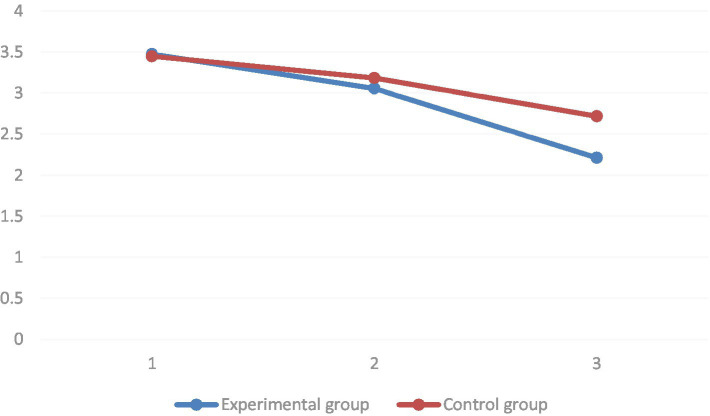
Comparison of the experimental and control groups before and after the experiment of the marginal mean of the interference mood dimension estimation.

Regarding the disturbed emotion dimension, it was found by multiple comparisons that the mean values of the disturbed emotion dimension of the pre-test, mid-test, and post-test decreased sequentially in the group introduced to the STEAM teaching concept. All of them reached the level of significance (*p* < 0.01). The mean values of the disturbed emotion dimension of the pre-test were significantly higher than those of the mid-test and post-test under the regular teaching intervention (*p* < 0.01). However, the estimated marginal mean values of the disturbed emotion dimension of the group taught with the introduction of the STEAM concept decreased significantly faster. The group simple effects analysis results showed that at baseline, there was no significant difference in the mean values of the disturbed emotions dimension between the two groups (*p* = 0.80). At the mid-test, there was also no significant difference between the two groups in the disturbed emotion dimension (*p* = 0.06). In the post-test, there was a significant difference between the mean values of the disturbed emotion dimensions of the two groups (*p* < 0.01) (as shown in Figure 12).

### The results of interviews with participants in the STEAM+ Shaolin staff teaching group

The qualitative insights from interviews shed light on students’ learning encounters within the STEAM-integrated Shaolin Staff course. Beyond the controlled experiment, this research also involved interviewing 20 participants from the experimental group regarding STEAM-related instructional aspects. The ensuing feedback highlights their perceptions regarding the fusion of science, technology, art, and math within the context of the Shaolin Staff curriculum.

Participant A stated, “I felt that science was integrated into the teaching and learning activities in the Shaolin Staff program. The teacher teaches techniques while analyzing the principles of human kinesiology and mechanics. This enabled me to understand better and optimize my staff movements. In addition, my teacher helps us analyze the staff’s effects on the body and study energy transformation and the role of force. If I lengthen the leverage of the staff, it will be more laborious for me to swing it.

Participant D said, “I had much fun using Mathematics in the Shaolin Staff course. By learning the angles and rotations of the staff, I began to realize the importance of mathematics in movement. I learned how to calculate angles and distances and apply geometric principles to improve my movements. This way of learning by combining math with actual movements has given me a new interest in math and helped me understand and apply math concepts more deeply in my Shaolin Staff studies.

Participant F said, “I appreciate using Art in the Shaolin Staff program. Through studying the history and culture of the Shaolin Staff, I began to appreciate and understand the artistic value of Shaolin Wushu. We learned physical expression and coordination in a class by imitating traditional Shaolin Staff movements and postures. While mastering my staff skills, I also developed my aesthetic ability. This way of incorporating artistic elements into my learning has given me a deeper interest in the Shaolin Staff and helped me develop my artistic perception.

Participant H said: I was pleasantly surprised by the use of Technology in the Shaolin Staff program. I could observe and evaluate my performance in real time through video analytics. This personalized feedback gives me a clearer understanding of my strengths and directions for improvement. At the same time, I improved my technical skills by learning to record and analyze my movements using technical tools. This way of learning by incorporating technology has increased my enjoyment of learning and made me more actively engaged in my studies.

Participant I said: For me, applying science, technology, art, and math to the Shaolin Staff course is a very integrated and interesting learning experience. Through studying these different disciplines, I have realized how interrelated and complementary they are. Science helps me to understand the principles of movement, mathematics helps me to calculate and optimize movements, art helps me to express and coordinate body movements, and technology helps me to observe and improve my performance. This integrated approach to the disciplines has enabled me to develop myself holistically and given me new insights for my future studies and life.

In summary, students felt positive about applying math, art, and technology to the Shaolin Staff curriculum. This integrated application enabled students to understand and apply concepts from related disciplines more deeply and stimulated their interest in learning. Through the parsing of scientific principles, mathematical calculations and optimization, artistic expression, and application of technology, students gained a rich learning experience and increased engagement in learning. They gained competence in several areas. This further validates the effectiveness of applying STEAM teaching concepts to the Shaolin Staff program and provides a new teaching model for physical education.

## Discussion and analysis

By applying STEAM teaching concepts and video analytics to the Shaolin Staff Technique course, students’ dimensions of learning engagement can be effectively improved. STEAM teaching methods emphasize students’ active participation and practical operation, making students the main body of learning, not just passive receivers (Madden et al., 2013). In this study, theoretical knowledge from different disciplines such as science, math, art, and physics is integrated into the teaching of Shaolin Staff, and the students not only master the Shaolin Staff technique itself but also learn the knowledge of other disciplines in the process of learning Shaolin Staff. In the process of learning the Shaolin Staff Technique, students not only mastered the technique itself but also learned other subjects. In addition to increasing students’ interest and participation in learning, it further stimulated their exploratory mentality and ability to think and solve problems. It can be seen that the application of STEAM education concepts in physical education can integrate physical education with other disciplines and enhance the effectiveness of physical education. This study is also a positive attempt to improve traditional Chinese physical education’s teaching concept and teaching effect. In studying the STEAM education concept to improve students’ learning interest and participation, some scholars combine the STEAM education concept with Olympic education. This improvement in education can make students have a stronger interest in Olympic-related knowledge and Olympic events and begin to actively pay attention to the Olympic movement and learn the Olympic spirit (Li and Yuan, 2022). Therefore, integrating STEAM education concepts into educational sessions can recognize the diversity of student learning, increase student engagement in learning, and promote integration and transformation across subject areas (Allina, 2018). In the Shaolin Staff Technique course, students can understand and master the techniques and principles more deeply, stimulate learning interest and motivation, and improve learning participation through actual practice and operation. Video analysis technology plays a key role in the Shaolin staff fighting course. By analyzing students’ movements through video, students can clearly understand their performance and progress in the learning process. Personalized feedback mechanisms motivate students and increase their commitment and engagement in learning. Students can observe and assess their movement skills, correct deficiencies, and see their progress. This personalized feedback boosts students’ self-confidence, motivation, and learning engagement (Cauley and McMillan, 2010).

At the same time, the combination of STEAM teaching methods and video analytics can also improve students’ aesthetic ability. Art is the foundation of all educational fields, and art education can break down the barriers between different educational fields by combining STEAM education concepts. It allows students to combine ideas and experiences to find new meanings, which develops their artistic creativity and aesthetic level and stimulates their aesthetic awareness and perception (Lee, 2014). The combination of art and science serves a unique purpose, enabling students to think outside the box to break down complex problems simultaneously. The corresponding solutions are then applied to the real world, providing a pathway to personal meaning-making and self-motivation (Land, 2013). Therefore, students are drawn to Shaolin culture while learning Shaolin Staff Techniques, thus developing and improving their aesthetic ability. This helps students appreciate and understand art and promotes the development of creative thinking and expression development.

Additionally, the analysis of technical movements in conjunction with physics demonstrates how this pedagogical approach improves student dimensions of learning engagement and athletic performance. Previous research on STEAM education has included integrating STEAM education with physics instruction. Creative integration education programs that utilize physical computing so that students can develop their creative problem-solving skills through computation, learn about science and other subjects in an integrated way, and increase students’ interest in learning (Yin et al., 2020). Incorporating STEAM education concepts into physics instruction can foster the development of students’ CT skills (Juškevičienė et al., 2021). Students can understand the scientific principles and mechanisms behind the movements by parsing the physics concepts of mechanics, kinematics, and kinetic energy transformation in movements. This theoretical knowledge increases students’ knowledge and understanding of technical movements, enabling them to grasp the key elements and execution of movements more accurately. Applying physics principles also gives students scientific thinking and analytical tools (Wang et al., 2015). Students can apply physics principles to analyze and evaluate their movement performance, analyzing factors such as the magnitude and direction of force changes in speed and acceleration and thus judging the accuracy and effectiveness of movements. In addition, through the guidance of the principles of physics, students can better master the essentials and techniques of movements and improve the quality of movement performance. This interdisciplinary approach aligns with education for sustainable development, which equips students with integrated knowledge and competencies to address sustainability issues (Annan-Diab and Molinari, 2017).

Generally speaking, the concept of STEAM education is being widely applied to teaching in various fields. Students can be exposed to the theoretical knowledge of other subjects while learning a certain subject, which in turn stimulates students’ desire for exploration and interest in learning. This educational concept has gradually become the development trend of modern education. Integrating the STEAM concept into physical education may become a new direction for future development. In this paper, it can be seen that applying STEAM teaching concepts and video analyzing technology to the Shaolin Staff Technique course can improve students’ participation in learning. The STEAM teaching method stimulates students’ interest and motivation through active participation and hands-on practice, and the video analysis technology provides personalized feedback to promote students’ self-confidence and engagement. At the same time, the analysis of technical movements in conjunction with the principles of physics enhances students’ understanding and knowledge of the movements and improves the accuracy of their performance. These mechanisms interact with each other to provide students with a rich learning experience and stimulate their learning interests and engagement. Therefore, integrating STEAM teaching concepts, video analyzing techniques, and physics principles is an effective teaching approach to improve students’ learning engagement and movement performance. While self-efficacy and self-directed learning are also important for student learning, this study narrowed its scope to assessing improvements in learning engagement. Further research could investigate how enhanced engagement may influence self-efficacy and self-directed learning abilities.

## Limitations

This study found that the experimental group had significantly better learning outcomes than the control group, which may be related to the application of STEAM pedagogy. However, we did not test the potential mediating variables such as interest in learning, sense of engagement, etc. The STEAM approach emphasizes hands-on learning and interaction, which may increase students’ interest and engagement and affect the learning outcomes. However, due to the study’s limitations, we were unable to measure and test these mediating variables, which is a limitation of this study. We believe STEAM pedagogy may affect learning outcomes through mediating processes, but further research is needed to confirm the exact mechanisms. In the future, we will improve the research design, collect data on the mediating variables, and test the mediating effects by using path analysis and other methods to explain more comprehensively the influence of STEAM teaching on learning outcomes. This study lays the foundation for further research and raises questions that deserve further investigation.

## Conclusions and recommendations

The study assessed how effectively the STEAM teaching model enhances student engagement in a Shaolin staff fighting course. Results reveal a significant increase in student learning engagement by blending science, technology, engineering, art, and math, along with video analytics for movement assessment and feedback. The utilization of a questionnaire to measure student learning engagement proved highly effective. This comprehensive approach not only nurtures interdisciplinary thinking but also aids in understanding intricate subjects such as sustainable development. Qualitative interviews provided evidence that the STEAM-integrated curriculum bolstered student motivation, knowledge application, skill enhancement, and overall learning experience in multifaceted manners.

Currently, China is actively promoting a strategy of cultural renaissance and advocating the modernization and transformation of traditional sports culture (Jinping, 2019). The teaching method explored in this study provides a way to improve traditional martial arts teaching. The Shaolin Wushu culture, as an important part of traditional Chinese culture, can improve students’ learning participation through this teaching method and indirectly promote the inheritance and innovation of Shaolin Wushu culture. This teaching method provides students with a richer learning experience, which can help them understand and master Shaolin staff skills more deeply, and at the same time, cultivates their interest in and identification with Shaolin Wushu culture.

This study brings the following implications for current physical education practices:

Firstly, the existing physical education teaching has the problems of single content and boring form, which cannot stimulate the student’s interest, and the diversity and comprehensiveness of the STEAM teaching method, as well as the intuitive image of video feedback, can make up for this shortcoming. Teachers should actively explore these new methods to make the curriculum more interesting.

Second, teachers need continuous professional development to improve their ability to utilize new technologies and theories. Educational administrations should provide teachers with training and resource support and encourage them to innovate the content and format of their teaching. At the same time, teachers need to upgrade themselves to better meet the diverse needs of their students.

Third, teachers should provide personalized instruction using video and scientific literacy to enhance student engagement during the teaching process. This requires educational institutions to create supportive environments that encourage teachers to explore new modes of teaching.

In conclusion, this study proves that the STEAM teaching method can effectively stimulate students’ interest in learning and promote the inheritance and innovation of traditional sports culture. The future development of physical education requires the joint efforts of teachers, schools, and administrations to improve the quality of teaching and create a supportive atmosphere for teaching innovations to increase students’ participation and promote the overall progress of physical education.

## Data availability statement

The original contributions presented in the study are included in the article/supplementary material, further inquiries can be directed to the corresponding author.

## Ethics statement

Ethical approval was not required for the studies involving humans because the project is limited to curriculum-related activities for instructional purposes. The studies were conducted in accordance with the local legislation and institutional requirements. Written informed consent for participation was provided by the participants or the participants’ legal guardians/next of kin.

## Author contributions

HC: Conceptualization, Funding acquisition, Investigation, Writing – original draft, Writing – review & editing. FR: Data curation, Methodology, Supervision, Funding acquisition, Writing – original draft. RC: Formal analysis, Project administration, Validation, Software, Writing – original draft. ZL: Resources, Visualization, Software, Writing – review & editing.
